# Content and Dynamics of Websites Shared Over Vaccine-Related Tweets in COVID-19 Conversations: Computational Analysis

**DOI:** 10.2196/29127

**Published:** 2021-12-03

**Authors:** Iain Cruickshank, Tamar Ginossar, Jason Sulskis, Elena Zheleva, Tanya Berger-Wolf

**Affiliations:** 1 Center for Computational Analysis of Social and Organizational Systems Carnegie Mellon University Pittsburgh, PA United States; 2 Department of Communication and Journalism University of New Mexico Albuquerque, NM United States; 3 Department of Computer Science The University of Illinois at Chicago Chicago, IL United States; 4 Translational Data Analytics Institute The Ohio State University Colombus, OH United States

**Keywords:** COVID-19, agenda setting, antivaccination, cross-platform, data mining of social media, misinformation, social media, Twitter, vaccinations, vaccine hesitancy

## Abstract

**Background:**

The onset of the COVID-19 pandemic and the consequent “infodemic” increased concerns about Twitter’s role in advancing antivaccination messages, even before a vaccine became available to the public. New computational methods allow for analysis of cross-platform use by tracking links to websites shared over Twitter, which, in turn, can uncover some of the content and dynamics of information sources and agenda-setting processes. Such understanding can advance theory and efforts to reduce misinformation.

**Objective:**

Informed by agenda-setting theory, this study aimed to identify the content and temporal patterns of websites shared in vaccine-related tweets posted to COVID-19 conversations on Twitter between February and June 2020.

**Methods:**

We used triangulation of data analysis methods. Data mining consisted of the screening of around 5 million tweets posted to COVID-19 conversations to identify tweets that related to vaccination and including links to websites shared within these tweets. We further analyzed the content the 20 most-shared external websites using a mixed methods approach.

**Results:**

Of 841,896 vaccination-related tweets identified, 185,994 (22.1%) contained links to specific websites. A wide range of websites were shared, with the 20 most-tweeted websites constituting 14.5% (27,060/185,994) of the shared websites and typically being shared for only 2 to 3 days. Traditional media constituted the majority of these 20 websites, along with other social media and governmental sources. We identified markers of inauthentic propagation for some of these links.

**Conclusions:**

The topic of vaccination was prevalent in tweets about COVID-19 early in the pandemic. Sharing websites was a common communication strategy, and its “bursty” pattern and inauthentic propagation strategies pose challenges for health promotion efforts. Future studies should consider cross-platform use in dissemination of health information and in counteracting misinformation.

## Introduction

Misinformation over social media contributes to the global growth in vaccine hesitancy. The World Health Organization (WHO) defined vaccine hesitancy as the “delay in acceptance or refusal of vaccines despite availability of vaccine services” [1] and declared it as one of the top 10 global health challenges in 2019, just before the outbreak of COVID-19 [2]. The “infodemic” that ensued as a response to the pandemic enhanced concerns about the rise of vaccine hesitancy in the 21st century [3] and about the key role that social media play in dissemination of vaccine-related misinformation [3]. Vaccine hesitancy discourse on social media does not represent just individual behavior. These messages are often part of concentrated disinformation efforts, intentionally promoted by nationalist right-wing politicians [4] and specific antivaccination leaders and “celebrities” [5,6]. Researchers also documented the efforts of foreign governments aiming to destabilize democratic processes by eroding the public trust in social institutions, including public health sources and the mainstream media [7-9]. With the COVID-19 pandemic, an increase was documented in the volume of misinformation on social media, including antivaccination propaganda [3,10-14]. Early in the pandemic, public health officials were concerned that the pandemic and the response to it led to worries about a global decrease in access to and acceptance of childhood and other vaccinations. In addition, public health efforts centered on developing a vaccine as a central strategy for ending the pandemic. Therefore, public trust in the safety and efficacy of vaccinations was considered paramount. Examining discourse on vaccination on social media is key to understanding public sentiments and to identifying the specific strategies used by different users, including antivaccination advocates, to erode trust in vaccinations.

Twitter has documented importance in setting the public and political players’ agendas [15], including in vaccinations [16,17]. According to agenda-setting theory [18], issues that are presented in the media frequently and prominently gain perceived salience by audiences. This salience is important as it impacts political agenda and policy making. According to Langer and Gruber (page 314) [19], “Unless an issue gets into the political agenda, it will not be discussed, debated in the legislature or acted upon by the government...news coverage is an important factor in making policy change more likely.” The theory was created to describe processes of *traditional media*, and specifically of legacy news. Traditional media, or *old media*, refer to the centralized mass media institutions that predated the information age, including print, television, radio broadcasting, studio-produced movies, and large advertising firms, among others [20,21]. In contrast to traditional media communication that was based on one-way technologies, new media are based on interactive and largely decentralized computer technologies [22], with the internet as the delineating telecommunication network [23].

Contrary to predictions about the death of traditional media in the age of the internet, and particularly following the rise of social media, studies indicate that they remain important. Traditional media have documented synergy with social media that amplify their mutual impact on the agenda in intermediate agenda-setting processes [19]. Research on the role of mass media during pandemics is fragmented, but a recent study that used computational methods in exploring the role of the media in covering pandemics revealed limited coverage of governmental health sources and frameworks [24]. This media coverage is important in influencing community behaviors. For instance, a study of COVID-19 coverage and behavior in Italy showed that the frames used by the news media influenced changes in community mobility significantly more than the effect of the number of daily death reports [25]. Moreover, whereas the most common source type for COVID-19 information seeking online was media outlets followed by governmental sources, governmental sources were the most likely to meet medical benchmark criteria for quality [26].

Studies on Twitter’s role in political agenda setting revealed an intermediate effect, in which the agendas of traditional media and Twitter were dissimilar, but exerted mutual influences [15]. However, past studies did not examine such processes in the context of vaccination-related tweets. Due to Twitter’s role in spreading health information and misinformation [27], understanding vaccination-related content and agenda-setting processes on this social platform can advance public health research and knowledge and inform future interventions.

Studies that used surveys to examine individual beliefs and intentions yielded important information on how audiences make sense of novel vaccines in the face of emerging pandemics, including their use of mental frameworks from previously known vaccines [28]. However, surveys are limited due to human recall and by access to participants. It is, therefore, pertinent to analyze the content and dynamics of social media that individuals create, share, and consume. New computational approaches to analyzing big data allow for analysis of communication about vaccination over social media in unprecedented ways [29-33].

Several studies documented the role of Twitter in disseminating vaccine-related misinformation prior to the current pandemic [8,34-38]. However, few studies examined patterns of this discourse. Notably, Twitter discourse about vaccination was reported as featuring heterogeneous conversations that were not dominated by particular subjects, sources, or users. Information sources that were tweeted frequently included health-specific sites, national media, medical organizations, and digital news aggregators [16]. A more recent COVID-19–related study [13] revealed that the largest single topic of Twitter conversations included comparisons between COVID-19 and influenza. Propagation of misinformation was observed in both previously known and new vaccine-opposing sources [13]. The study established that known sources of vaccine communication continued to engage in the topic early in the pandemic. However, it was limited to analysis of tweets that were tweeted over the course of one day. To get a more holistic picture of these conversations, it is important to examine communication about vaccination as part of Twitter’s COVID-19 discourse over extended time frames.

An additional lacuna in research on vaccine-related communication on social media involves its focus on single social media platforms. Research documented that most social media users use multiple media sources and social media platforms [39], and often go back and forth between different platforms [40]. It is, therefore, important to explore cross-platform use. Analysis of links to websites and the website domains that are shared on tweets can provide information about such cross-platform use and spread of information sources. Specifically, including a URL in a tweet allows readers to link to the website. For instance, most tweets that responded to misinformation with cross-platform links during Hurricanes Harvey and Irma focused on debunking misinformation and used news source URLs in their response [41]. Hence, such cross-platform use can serve to share information during a time of crisis. Examination of website sharing in COVID-19 conversations documented both the importance of traditional news sources and the propensity for virality of low-quality sources. Whereas low-quality information sources were tweeted at higher rates compared to high-quality health sources, traditional news sources were shared at a much higher rate than other sources [42]. Moreover, shared websites within COVID-19 Twitter conversations revealed users’ political stance [43], thus lending more support to the close links between health and political debates during the pandemic.

These previous studies underscored the potential importance of cross-platform information sharing on Twitter. Analyzing both the URLs and the domains shared as external content on Twitter can provide insights into the type of specific content and information sources included in social media messages about vaccination. Despite this importance, vaccine-related cross-platform use over Twitter received limited scholarly attention. Examination of links to websites shared within vaccination-related tweets early in the COVID-19 pandemic can enrich knowledge by gaining a broader understanding of this communication. Empirical implications of this knowledge include informing strategies for evidence-based vaccine-related message dissemination over social media. Moreover, it can shed light on the role of traditional media in the era of social media, as well as on how social media are used in cahoots with vaccine-related communication and their dynamics over time. Finally, in view of the documented role of inauthentic propagation of vaccine-related content on Twitter [8,37] and in COVID-19–related discussions [44], studies should go beyond typologies of vaccine-related content [45] and understand strategies employed in the spread of this content. New social cybersecurity methods [46] can aid in such examinations.

The goal in this study was to examine website sharing in vaccine-related tweets posted to COVID-19 conversations in the 20 weeks following the declaration of the pandemic. Specifically, we analyzed tweets that were posted from February 1 (two days after the WHO declared the outbreak of COVID-19 to be a Public Health Emergency of International Concern) through June 23, 2020, and sought to examine the magnitude, temporal patterns, and content of websites shared within these tweets. This study will provide unique contributions to theory and practice. Our examined time frame took place prior to the development of the vaccine and the implementation of COVID-19 vaccination campaigns. Therefore, tweets posted during that period can indicate the degree to which vaccinations were included in COVID-19–related discourse from its inception. It will also reveal the information sources that were promoted through cross-platform link sharing. These findings have the potential to indicate the effectiveness of official health sources in leading the agenda as health information providers and the prominence of vaccine-opposing sources. It can also uncover some of the tactics of the vaccination-opposing movement over time and in response to this new, unexpected, global health threat. This understanding is important for advancing theories about the role of social media in public health crises, as well as for informing future policies, interventions, and dissemination of health information to address audiences’ informational and emotional needs.

Given the importance of vaccination-related discourse and website sharing within COVID-19 Twitter conversations, including an understanding of the sources of vaccination-related information and their spread, we posed the following research questions.

First, as this is, to our knowledge, the first study to examine external content sharing in the context of vaccination in early COVID-19 conversations, we were interested in understanding the magnitude of external content, the degree to which vaccinations were featured in conversations about COVID-19 early in the pandemic, and the prevalence and dynamics of website sharing within these tweets.

Therefore, we posed the first research question: What are the prevalence and dynamics of vaccination-related tweets, including website sharing, posted between February 1 and June 23, 2020, as part of COVID-19 conversations, as evident in the number of these tweets over time?

We were further interested in learning about agenda-setting processes that are demonstrated in this relatively new social media strategy of information source promotion. As websites’ domains, such as television networks, vlogs, or individual social media accounts, represent specific information sources, we aimed to learn about these sources and their characteristics. Specifically, in view of the importance of information sources in public health communication, we sought to identify the sources of the websites that were shared most prominently in the early months of the pandemic in tweets about vaccination that were posted to COVID-19 Twitter conversations.

Therefore, the second research question was posed: What are the characteristics of the 20 most-shared website domains?

In addition to the information sources, we were interested in exploring the content of the most-shared information, as evident in the 20 most-tweeted websites in our data set. The prominence of these websites can stem from users being activated by the content and their desire to share it. However, specific spread strategies and coordinated efforts might also drive this prominence. Therefore, we wanted to examine both the content of the websites and the specific information that was shared in this cross-platform modality, as well as their propagation.

Therefore, the third research question was posed: What characterizes the content and spread of the 20 most-shared websites?

## Methods

### Data

The analysis encompassed two different data sets of COVID-19–related tweets. The first data set was based on a collection of tweet IDs gathered using general COVID-19–related keywords, such as “coronavirus” and “Wuhancoronavirus” [47]. We used “hydration” [48], a process of gathering all the pertinent information about each tweet into the JSON format file [49] via the Twitter search application programming interface (API) [50], on all of these tweet IDs. This process only populated data from tweets that were available on Twitter at the time of hydration and would exclude banned users or deleted tweets. This data set had around 1 million tweets. The second data set included approximately 4.5 million COVID-19–related tweets collected from January 29 to June 23, 2020, using Twitter’s streaming API [51,52]. Since these tweets were collected in a streaming fashion, as they were tweeted, it allowed for analysis of some tweets that were otherwise no longer available on Twitter.

**Figure 1:**
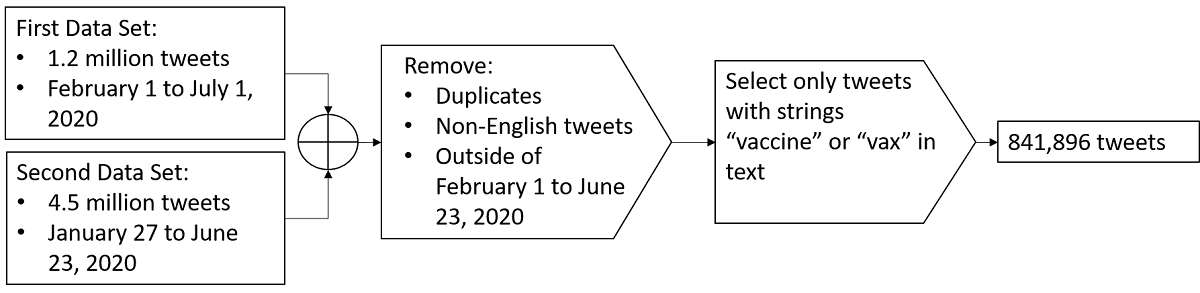
Data set combination, filtering, and exclusion process.

Both data sets were then filtered to include only the dates of overlap (February 1 to June 23, 2020) and to remove any duplicated tweets across the data sets. We then filtered the data to include only English-language tweets. Given our interest in tweets about vaccination, each data set was filtered using the substrings “vax” and “vaccin.” This process ensured that the tweets included in our analysis referred to vaccinations. The resulting data set contained 841,896 English-language tweets. Since our focus was on analyzing content available to users rather than the identity of users, we did not attempt to distinguish between human users and machine accounts (ie, bots) [53]. Figure 1 displays a graphical flowchart for the data selection and exclusion process.

### URL Extraction

We first extracted the website URLs from the JSON “entities” object of each tweet in order to get the original URL rather than the version automatically shortened by Twitter. URLs that were still shortened were unshortened to their original form using an API [54]. To preprocess the URLs for analysis, we then removed all URL query terms from all domains.

### Content Analysis

In addition to computational methods, mixed methods content analysis was conducted in analyzing the 20 most-tweeted websites to identify the source of the websites and their content. First, the sources of the 20 most-tweeted URLs and the dates they were posted were recorded. The content of the 20 most-tweeted URLs was coded using inductive qualitative methods using the constant comparative method [55-57]. This design, which prioritized quantitative methods, is consistent with an *explanatory sequential* design. This design involves implementing quantitative research methods first, followed by qualitative methods, with the aim of explaining the quantitative methods [58]. Mixed methods research is particularly appropriate in studying complex social phenomena, and this approach was appropriate, as the quantitative approach was not deemed sufficient [58-60]. The qualitative research approach is suitable for exploratory studies when researchers are unable to use theory to produce hypotheses or theoretical-driven prediction [61].

The qualitative analysis followed a multistep iterative process. A coauthor with expertise in mixed methods research created initial codes and recorded memos. The initial coding involved line-by-line detailed reading of the data, aimed at understanding the different views and actions described in the different URLs and approaching coding in an inductive manner while remaining open to different potential theoretical directions emerging from the data [62]. During the second phase of the analysis, focused coding was conducted. The focused coding entailed coding of the significant and frequent themes that emerged during the initial coding. Focused coding was helpful in synthesizing and conceptualizing the data and the research [62], while also remaining cognizant of the different sources generating the content. Comparisons of statements and incidents were noted within and across the different URLs. At the second stage, previous research and categorizations were considered in addition to the texts at hand, and these informed the categories of the URLs’ framing. The first category included content that overtly advanced doubts about at least one of the following: vaccines’ efficacy, vaccines’ safety, and the motives of those who fund, develop, and/or test them (ie, vaccine-opposed). Conversely, the second category captured content that featured the efficacy and/or safety of COVID-19 vaccines. The third category included content that focused on advances in development of vaccines, including news on the development of specific vaccines and related scientific breakthroughs. In this third theme, coders also annotated whether the advancements that were reported were based on meaningful developments or whether they reflected anecdotal information and unfounded claims. The fourth category related to content that highlighted political aspects of vaccination, including portraying political processes as influencing vaccine development and availability to the public. This political content was further coded to capture whether vaccination was, in fact, the focus of the overall content. In addition, the coders noted whether content in any category could have increased distrust in vaccination by using implicit cues that casted doubts on the integrity of the process, those developing vaccines, or decisions and decision makers. For instance, a news story that announced that a COVID-19 vaccine was developed in 3 hours was coded as including “vaccine-opposing” sentiment, as it was judged to be increasing concerns about a vaccine that was developed so rapidly and, therefore, likely to reduce trust in its safety and efficacy.

In the third stage of the qualitative analysis, the overall theme of “politicizing vaccination” emerged. A graduate student with training in qualitative research followed this process independently by coding each of the 20 URLs using inductive coding first and then coding by the previous categories. They then provided a quotation from each URL to support the coding. As a final check on consistency of results, we evaluated the intercoder agreement for all of the URLs between the initial coding and the third stage of qualitative analysis. The use of constant comparative analysis [62] helped us develop our analytical categories, including attention to contradictions.

### Twitter Spread Analysis

To examine the particular Twitter spread strategies that were used to propagate the most-tweeted websites, we have applied social cybersecurity methods to identify coordinated link sharing and flooding (or spamming) of the websites by tweeters [46,63]. Specifically, we analyzed all the tweets that shared the top 20 most-tweeted websites. First, we excluded retweets and removed the following text from the remaining original tweets: mentions, URLs, trailing white space, and formatting characters (ie, “\n”). Then, we recorded the number of tweets that included a URL to each website, unique users that tweeted the website, unique texts across all of the tweets that contained the website, tweets that featured a website that were tweeted within an hour of the first tweet of that same website, and nonreply mentions, as well as the range of days between the first and last tweet that included the website.

## Results

### Overview

**Figure 2:**
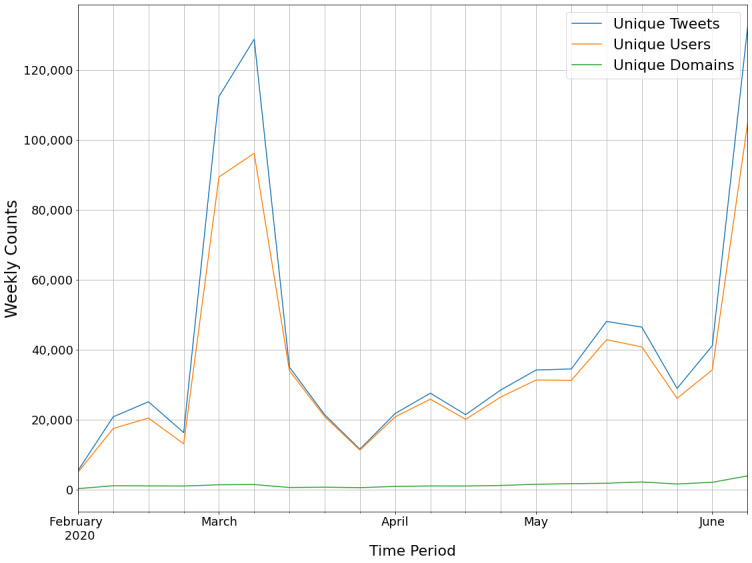
Counts of unique tweets, users, and website domains for all of the vaccination tweets.

Our aims were to understand the magnitude, dynamics over time, content, sources, and spread of websites shared within vaccine-related tweets as part of COVID-19 Twitter conversations. The first research question centered on the prevalence and dynamics of vaccination-related tweets, as well as website sharing posted as part of COVID-19 conversations over time. The analysis revealed that these conversations demonstrated an overall growth in tweets that related to vaccination. It also showed that website sharing had distinct patterns compared to overall tweets about vaccination. As mentioned above, our data set contained a total of 841,896 tweets. As seen in Figure 2, tweets about vaccination spiked in March and again in June. A corresponding spike in tweets that were retweeted was observed in March. In contrast, a more modest increase was observed in March in website sharing, followed by a leveling in April. In addition, sharing websites and the diversity in unique websites shared increased over time, indicating that Twitter users spread more external content from a greater number of sources as the scope and scale of the pandemic increased in the early months of the pandemic.

**Figure 3:**
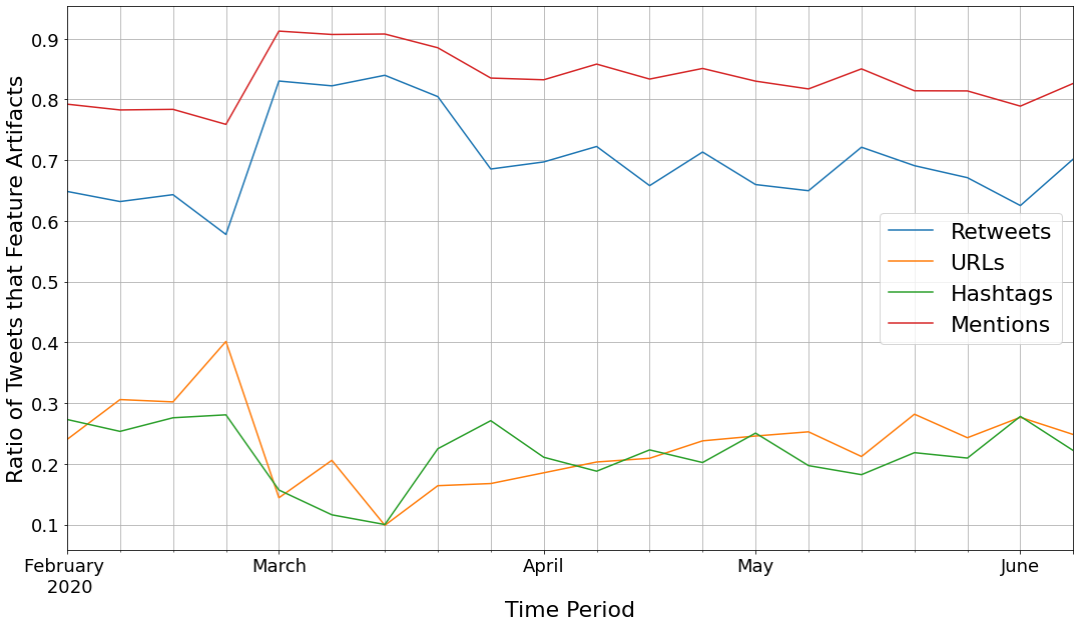
Rates of retweets, hashtags, mentions, and website sharing in all of the vaccination tweets.

Our analysis revealed that 1 in 5 of the 841,896 tweets (n=185,994, 22.1%) contained at least one website. A total of 1 in 4 of the 524,998 users (n=128,408, 24.5%) tweeted at least one website. In comparison, only 19.4% (n=163,743) of all tweets contained at least one hashtag, and 23.0% (n=120,699) of users tweeted at least one hashtag. Additionally, 85.2% (n=717,150) of all tweets contained a mention of another user’s account (ie, using the “@” symbol to refer to another Twitter account), and 87.4% (n=459,038) of users tweeted at least one mention in a tweet. Of the mentions, only 12.1% (n=87,097) were replies (ie, when a user was directly replying back to the tweet of another user). Figure 3 displays the rates of usage for these different social media artifacts.

A total of 1 out of 5 of the tweets that mentioned vaccination (185,994/841,896, 22.1%) included links to websites. These websites included 11,311 unique website domains. Most domains (n=6962, 61.6%) were tweeted with only one unique website tweeted.

### Analysis of Website Domains

Research question 2 explored the 20 most-shared website domains, with a focus on the role of legacy media, social media, and public health sources. As displayed in Table 1 [64-83], the majority (n=14, 70%) of the 20 most-tweeted website domains consisted of traditional news media, including news organizations, newspapers, and television networks. However, the most-tweeted website domain was Raw Story, a US online tabloid that is classified as progressive. Another tabloid, the New York Post, was ranked as the 19th most-tweeted website domain. The only social media platform included was YouTube, which was the third most-tweeted domain. Similarly, the only official governmental and/or health source was the US Centers for Disease Control and Prevention (CDC), which was ranked as the 20th most-tweeted website domain.

Websites associated with these 20 domains constituted 8.25% (n=6244) of all of the 75,642 websites tweeted. Two of the most-tweeted website domains—Raw Story and The Jerusalem Post—had most of their tweets from just one news story each. Notably, the top two most–individually tweeted websites were in this category, indicating that “viral” tweets can increase a domain’s popularity.

On average, each domain had 4.26 (SD 24.4) websites per domain, with a mode of 1 website, indicating that many of the domains only ever had one website associated with them. In total, 62.7% (n=6963) of all domains in the data had only one unique website associated with that domain. An exception was YouTube, which had the greatest number of unique websites of any domain, followed by the CDC website.

**Table 1:** Top 20 most-tweeted website domains.

Website domain	Tweets, n	Percentage of all tweets with website URLs that originate from website domain, %	Unique websites per domain^a^, n	Type of domain and country of origin
Raw Story [64]	13,261	7.1	101	US online tabloid
Reuters [65]	4347	2.3	577	International news organization
YouTube [66]	4106	2.2	2013	International, US-based social media platform
The Guardian [67]	3167	1.7	364	UK newspaper
The Jerusalem Post [68]	3140	1.7	92	Israeli newspaper
Bloomberg [69]	2374	1.3	173	International, US-based news agency
CNBC [70]	2161	1.2	282	US television channel
The Daily Mail [71]	2102	1.1	291	UK newspaper
CNN [72]	1888	1.0	302	Multinational, US-based television channel
The New York Times [73]	1716	0.9	332	US newspaper
The Cable [74]	1642	0.9	19	Nigerian digital newspaper
STAT [75]	1552	0.8	122	Health-oriented US news website
Business Insider [76]	1447	0.8	203	US financial news website
The Washington Post [77]	1348	0.7	209	US newspaper
BBC [78]	1320	0.7	106	UK public service broadcast organization
Sky News [79]	1301	0.7	147	UK television news channel
The Independent [80]	1280	0.7	172	UK newspaper
The Hill [81]	1270	0.7	152	US newspaper
New York Post [82]	1148	0.6	152	US conservative-leaning tabloid
Centers for Disease Control and Prevention [83]	1139	0.6	435	US government health organization

^a^This is the number of unique websites that originate from the higher-level domain. For example, a news website can have several unique websites representing different news stories that all come from the same single news website domain.

**Figure 4:**
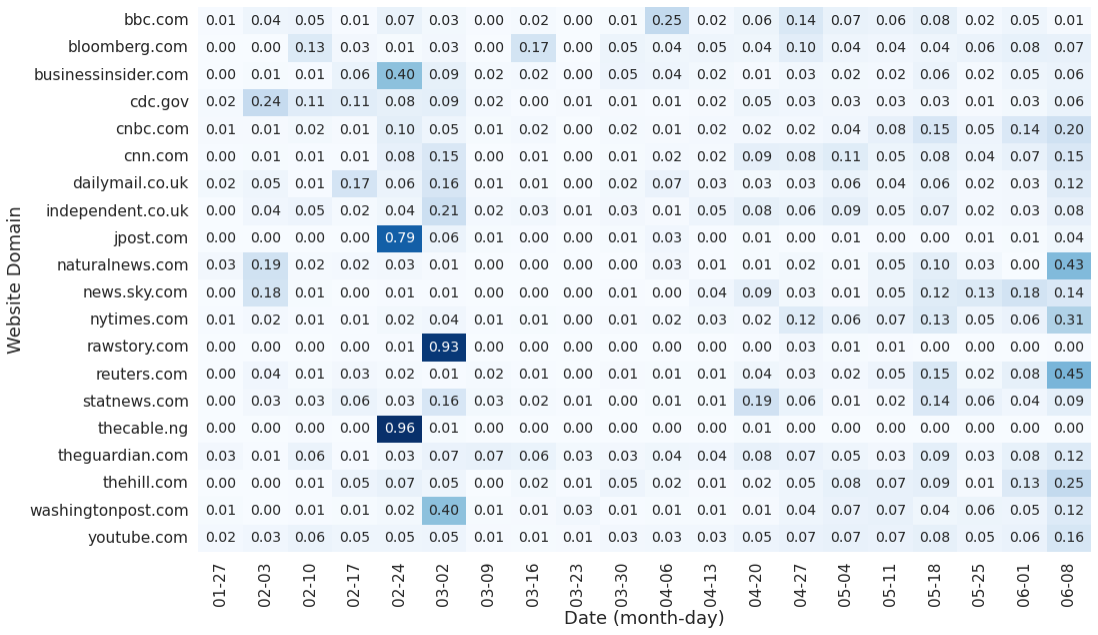
The 20 most-used domains overall by the number of tweets featuring each domain. Counts are normalized within each time period (week).

The temporal analysis of the patterns of the 20 most-tweeted domains documented three distinct peaks of domain usage, as presented in Figure 4. The peaks included The Jerusalem Post and The Cable from February 20 to 24 and Raw Story on March 4. These spikes in domain usage were the result of tweeting a particular website from that domain. Specific stories that became “viral” underlined domain activity. Tweeted domains were typically shared over one week, or even one day. This pattern can, therefore, be characterized as “bursty,” as opposed to having different websites associated with specific domains tweeted consistently across longer periods of time.

In contrast to the majority of the domains that were characterized by “bursty” activity, some domains, such as YouTube and The Guardian, had consistent usage over time. The 20 most–persistently tweeted domains are summarized in Table 2 [65-67,69-73,75-78,80,82-88].

As Table 2 shows, YouTube was the most–persistently tweeted domain. Out of the 20 domains, the 12 (60%) that were tweeted most persistently over time were news organizations, and 2 (10%) included official US federal health organizations. Both the CDC and the National Institutes of Health (NIH) were included in this list, indicating the persistent sharing of content of these domains. Similarly, in addition to YouTube, Instagram was a second social media platform that made the list, as well as Google.

**Table 2:** The 20 most–persistently tweeted website domains.

Home domain	Type of domain	Days that a website from the domain was tweeted at least once, %
YouTube [66]	Social media platform	77
The Guardian [67]	UK newspaper	76
The Daily Mail [71]	UK newspaper	72
Centers for Disease Control and Prevention [83]	US federal health organization	71
CNBC [70]	US television news channel	71
The New York Times [73]	US newspaper	70
Reuters [65]	International news agency	70
Bloomberg [69]	US business newspaper	67
STAT [75]	Health-oriented US news website	66
Business Insider [76]	US financial news website	65
Instagram [84]	Social media platform	65
The Independent [80]	UK newspaper	65
CNN [72]	Multinational, US-based television channel	64
Google [85]	Multinational technology company	64
New York Post [82]	US tabloid	63
BBC [78]	UK public service broadcast organization	61
MSN [86]	Web portal by Microsoft	61
The Washington Post [77]	US newspaper	61
BBC [87]	UK public service broadcast organization	60
NCBI (National Center for Biotechnology Information), NIH (National Institutes of Health) [88]	US federal health organization	59

### Analysis of Websites

The third research question explored the content and dynamics of spread of the 20 most-tweeted websites. Table 3 [89-108] displays the 20 most-tweeted individual websites within the data.

The 20 most-tweeted websites comprised around 13% of all the tweets containing websites (n=185,994) in the data set. The most-tweeted website, which was by Raw Story, accounted for almost half of these, with 6.6% (n=12,201) of the overall number of tweets containing websites. As Table 3 indicates, the majority (n=11, 55%) of the 20 most-tweeted websites were tweeted by traditional media sources or news organizations, and the sources of 6 other websites (30%) were tabloid and digital-only newspapers and websites. One fake news website, a petition website for right-wing causes, and another domain were the sources of three more websites. The latter two were the only nonnews sources among the top 20 most-tweeted websites.

As an overarching theme, the qualitative analysis indicated that the content of these websites demonstrated politicization of vaccination. The single most-tweeted website was by the digital tabloid Raw Story, which described Republicans blocking the COVID-19 bill to avoid posing limits on pharmaceutical companies’ charges for the vaccine. This politicization of vaccination was evident in other websites. As seen in Table 3, 9 out of 20 (45%) websites (#1, #4-6, #8, #9, #15, #17, and #20) focused on political aspects of the vaccine and its development. In 2 of them (#6 and #15), vaccines were mentioned merely as a minor issue. Moreover, 2 of these featured cues that could increase, or were related to, distrust in vaccination. These included a Fox Business story (#14) claiming that a vaccine was developed in 3 hours in collaboration with China and was funded by the Gates Foundation, and a BBC News story (#18) that reported on comments by a top health official in France that called to test the COVID-19 vaccine in Africa. Although the WHO was cited as denying these comments, the story, overall, featured distrust in vaccine development, provided direct citations of celebrities that responded to the possibility of testing in Africa, and referred to controversial testing of HIV medications on prostitutes in Africa. Similarly, the STAT story (#20) that covered Dr Rick Bright’s departure from directing the Biomedical Advanced Research and Development Authority, without stating a reason for his departure, could have led audiences to have reduced trust given their portrayals of plots that left much to the imagination. Other frames included criticism of then-President Trump’s competence in combating the pandemic following comments or decisions that he made, including suggesting using a flu vaccine to prevent COVID-19 (#8 and #15) and pulling out of a national effort to speed vaccine development (#17).

**Table 3:** The 20 most-tweeted websites.

Rank	Title of webpage	Source; type	Topic	Coding	Tweets, n	Date in 2020
1	GOP blocking coronavirus bill — because it limits how much drugmakers can charge for a vaccine: Report [89]	Raw Story; US progressive-leaning tabloid	Republicans block coronavirus bill because it limits how much pharmaceutical companies can charge	4. Political focus: the main focus is on the political processes and motives related to vaccination	12,201	March 3
2	Israeli scientists: 'In a few weeks, we will have coronavirus vaccine' [90]	The Jerusalem Post; Israeli newspaper	Israeli scientists are close to developing a COVID-19 vaccine	3b. News on vaccine development that proved unfounded	2780	April 13
3	Israeli researchers announce breakthrough on coronavirus vaccine [91]	The Cable; Nigerian online newspaper	Israeli scientists are close to developing a COVID-19 vaccine	3b. News on vaccine development that proved unfounded	1507	February 9
4	EU sets out plans for advance orders of coronavirus vaccines [92]	The Irish Times; Irish newspaper	Britain will not be included in European COVID-19 vaccine supplies	4. Political focus: the main focus is on the political processes and motives related to vaccination	865	June 11
5	UK will not participate in EU’s coronavirus fast track vaccine scheme [93]	The New European; UK pro-Europe newspaper	Britain will not be included in European COVID-19 vaccine supplies	4. Political focus: The main focus is on the political processes and motives related to vaccination	789	June 12
6	James Clapper refuses to testify to Congress in person ‘until there’s a COVID vaccine’ [94]	True Pundit; US fake news website	Former Director of National Intelligence refused to testify until there is a vaccine	4. Political focus: the main focus is on the political processes and motives related to vaccination	530	May 15
7	AstraZeneca agrees to supply Europe with 400 million doses of COVID-19 vaccine [95]	Reuters; international news organization	AstraZeneca signed a contract to supply 400 million doses of COVID-19 vaccine to Europe	3a. News on vaccine development that proved founded	521	June 13
8	Read Pedagogy of the Oppressed by Paulo Freire (tweet by Joshua Potash, liberal leaning with 146,000 followers) [96]	Twitter; US-based social media platform	“Trump thinks we should use the flu vaccine to defend against coronavirus. We could not be in worse hands.”	4. Political focus: the main focus is on the political processes and motives related to vaccination	512	March 2
9	Trump’s baffling coronavirus vaccine event [97]	Washington Post; US newspaper	Commentary on Trump’s interactions with vaccine makers	4. Political focus: the main focus is on the political processes and motives related to vaccination	454	March 3
10	PETITION: No to mandatory vaccination for the coronavirus [98]	Life Petitions; petitions website	Petition to prevent mandatory COVID-19 vaccination	1. Content that overtly advances doubts regarding vaccines’ efficacy and safety and the motives of those who fund, develop, and/or test them	434	January 23
11	Meet the all-female team working to create a COVID-19 vaccine in Maryland [99]	WJLA: ABC News; local DC news affiliate	Team working on developing COVID-19 vaccine	3b. News on vaccine development that proved unfounded	428	February 28
12	US scientists have completed a coronavirus vaccine, Texas-based genetic engineering company claims [100]	The Daily Mail; UK newspaper	Texas-based scientists reported completion of COVID-19 vaccine development	3b. News on vaccine development that proved unfounded	391	February 20
13	Israeli scientists: 'In a few weeks, we will have coronavirus vaccine' [101]	The Jerusalem Post; Israeli newspaper (mobile version)	Development of COVID-19 vaccine in Israel	3b. News on vaccine development that proved unfounded	387	April 13
14	California lab says it discovered coronavirus vaccine in 3 hours [102]	Fox Business; US television channel	Development of COVID-19 in California in 3 hours, funded by Gates, with China	3b. News on vaccine development that proved unfounded	381	February 13
15	Experts baffled as Trump asks why they can't just use flu vaccines to prevent coronavirus [103]	Indy100; UK online newspaper	Experts are baffled at Trump suggestion to use flu vaccine	4. Political focus: the main focus is on the political processes and motives related to vaccination	377	March 3
16	COVID-19 vaccine shipped, and drug trials start [104]	TIME; US news magazine	Moderna vaccine shipped, and trials start	3a. News on vaccine development that proved founded	363	February 25
17	Trump removes US from global initiative to develop coronavirus treatments and vaccines [105]	Raw Story; US tabloid	Trump removes United States from global initiative to develop COVID-19 treatment	4. Political focus: the main focus is on the political processes and motives related to vaccination	348	February 25
18	Coronavirus: Africa will not be testing ground for vaccine, says WHO^a^ [106]	BBC News; UK broadcast organization	WHO says Africa will not be a testing ground for COVID-19 vaccine	3b. News on vaccine development that proved unfounded	325	April 6
19	Here's why Obamacare would likely make any coronavirus vaccine free for patients — and prove critical in fighting the disease [107]	Business Insider; US online media company	COVID-19 vaccine will be available for free thanks to the Affordable Care Act	2. Provaccination: the content focuses on the efficacy and/or safety of COVID-19 vaccines, as a way to end the pandemic	319	February 29
20	Director of US agency key to vaccine development leaves role suddenly amid coronavirus pandemic [108]	STAT; US health news website	Rick Bright steps down	4. Political focus: the main focus is on the political processes and motives related to vaccination	300	April 21

^a^WHO: World Health Organization.

Of the 9 (45%) websites that covered news about advances in vaccine development, only 2 of the developments that were covered proved founded (#7 and #16 covered AstraZeneca and Moderna, respectively). TIME reported on the rollout of Moderna vaccine clinical trials (#16), stood out as the only website that provided medical framing of the content, and included explanations regarding the vaccine’s mechanism of using messenger RNA (mRNA). In contrast, 7 (35%) stories covered advancements that, at the time of analysis, were unfounded or did not come to fruition (#2, #3, #11-14, and #17). Notably, 3 of these included news on development of an Israeli vaccine that allegedly was 3 days away from the finish line and 90 days from approval. This information came from the Israeli Science and Technology Minister, and the sources were an Israeli newspaper in English and a Nigerian digital newspaper. Of the coverage of vaccine development that did not reach the market, only the report about the Novavax vaccine (#11) included information about the phase of the trial. The report on Greffex (#12) included some scientific information on the technology of the vaccine and a timeline that emphasized the lengthy process.

Only 1 (5%) website expressed explicit opposition to vaccination (#10). It consisted of a petition to block mandatory vaccination, which was included in a website domain that promoted petitions for conservative, right-wing causes. Similarly, only 1 (5%) website (#19), by Business Insider, a US online media company, provided provaccination framing by positioning vaccines as the way to end the pandemic.

Table 4 [89-108] presents information on the original tweets that included each of the 20 most-tweeted websites.

**Table 4:** Tweets spreading the 20 most-tweeted websites.

Title of webpage	Website	Unique tweets with URL, n	Unique texts of tweets, n	Unique users that tweeted URL, n	Mentions in tweets with URL, mean (SD)	Retweets of tweets with URL, n	Days between first and last tweet of URL, n	Tweets within first hour of first tweet, n
GOP blocking coronavirus bill — because it limits how much drugmakers can charge for a vaccine: Report [89]	Raw Story	838	437	786	0.31 (1.61)	11,363	12	129
Israeli scientists: 'In a few weeks, we will have coronavirus vaccine' [90]	The Jerusalem Post	710	352	612	0.33 (1.14)	2070	78	20
Israeli researchers announce breakthrough on coronavirus vaccine [91]	The Cable	31	20	26	0.71 (1.54)	1476	3	4
EU sets out plans for advance orders of coronavirus vaccines [92]	The Irish Times	78	41	77	0.21 (0.43)	787	2	1
UK will not participate in EU’s coronavirus fast track vaccine scheme [93]	The New European	50	34	48	0.08 (0.3)	739	2	2
James Clapper refuses to testify to Congress in person ‘until there’s a COVID vaccine’ [94]	True Pundit	20	13	20	0 (0)	510	21	1
AstraZeneca agrees to supply Europe with 400 million doses of COVID-19 vaccine [95]	Reuters	62	38	57	0.21 (0.57)	459	1	5
Read Pedagogy of the Oppressed by Paulo Freire [96]	Twitter	112	111	110	0.16 (0.49)	400	2	7
Trump’s baffling coronavirus vaccine event [97]	The Washington Post	351	197	339	0.19 (0.75)	103	66	19
PETITION: No to mandatory vaccination for the coronavirus [98]	Life Petitions	241	90	156	0.50 (2.32)	193	37	3
Meet the all-female team working to create a COVID-19 vaccine in Maryland [99]	WJLA: ABC News	56	33	56	0.16 (0.49)	372	18	2
US scientists have completed a coronavirus vaccine, Texas-based genetic engineering company claims [100]	The Daily Mail	135	64	133	0.67 (0.75)	256	26	22
Israeli scientists: 'In a few weeks, we will have coronavirus vaccine' [101]	The Jerusalem Post (mobile version)	110	55	104	0.79 (3.99)	277	51	1
California lab says it discovered coronavirus vaccine in 3 hours [102]	Fox Business	225	123	215	0.24 (0.69)	156	19	8
Experts baffled as Trump asks why they can't just use flu vaccines to prevent coronavirus [103]	Indy100	5	2	4	0.2 (0.4)	372	0	3
COVID-19 vaccine shipped, and drug trials start [104]	TIME	111	81	109	0.43 (1.16)	252	63	1
Trump removes US from global initiative to develop coronavirus treatments and vaccines [105]	Raw Story	17	13	17	0.52 (1.24)	331	1	3
Coronavirus: Africa will not be testing ground for vaccine, says WHO [106]	BBC	18	14	17	0.38 (1.16)	307	1	2
Here’s why Obamacare would likely make any coronavirus vaccine free for patients — and prove critical in fighting the disease [107]	Business Insider	230	127	222	0.64 (1.05)	89	6	4
Director of US agency key to vaccine development leaves role suddenly amid coronavirus pandemic [108]	STAT	24	11	24	0.58 (0.57)	276	6	6

The Twitter propagation statistics of some of the websites show attempts at spreading the websites through inauthentic means. Specifically, a high percentage of the tweets that included links to The Cable website (#3) and to the STAT website (#20) had a high percentage of retweets only, an indicator of spreading by flooding or spamming of a website on a social media platform in an effort to get the website artificially trending and, consequently, to give more exposure to other social media users. In addition, the text of over half of the tweets that included the 20th most-tweeted website, STAT, was identical, which is indicative of coordinated, inauthentic link sharing. Evidence of such coordinated inauthentic link sharing was also present in tweets that included links to the Life Petitions website (#10), which also had relatively few unique tweet texts compared to the number of tweets of the website and the number of unique users that tweeted that website.

## Discussion

### Principal Findings

This study is the first to examine the prevalence, dynamics, and content of websites shared in vaccination-related tweets. We focused on tweets that were part of COVID-19 conversations over 20 weeks, following the WHO’s announcement of COVID-19 as a pandemic until June 23, 2020. The main finding of this study is the use of a cross-platform strategy for promoting politicization of COVID-19 vaccination well before the rollout of these vaccines. This politicization of content, promotion of unfounded “advancements” in vaccine development, and coverage of unsettling political plots that left much unexplained are likely, in turn, to contribute to a decrease both in the public’s knowledge of the science behind vaccine development and effectiveness and its trust in vaccination. Future studies should investigate the impact of exposure to this coverage.

Whereas previous research on the topic typically focused on the degree to which Twitter discussions reflected specific vaccine sentiments, our study indicates the politicization of the topic, which was shared by both progressive-leaning sources and content (eg, the Raw Story online tabloid) and legacy media (eg, The Washington Post) as well as by right wing–leaning sources, including legacy media (eg, The Jerusalem Post) and fake news sources (eg, True Pundit).

The websites that were tweeted represented diverse communication sources, with traditional news media making the top shared domains. Both the prominence of legacy news media in websites shared and the emergence of nontraditional media outlets, such as tabloids, vlogs, and other social media, exemplify processes of intermediate agenda setting in the new media environment. These processes were previously documented in political content [15], and this study extends them to this health context. Along with “rehashing” legacy news content, as was evident in the majority of the sources shared, Twitter has also given rise to nontraditional, digital-only content. These nontraditional sources typically reached salience in terms of website sharing when a story they published became viral. For instance, Raw Story, a digital tabloid [109], featured the most-tweeted website in its story of Republicans blocking a bill in order to protect pharmaceutical companies from limitations on vaccine-related profits. The salience of nontraditional sources demonstrates an intermedia agenda-setting process that provides a platform for individuals who were previously blocked from entering the elite spaces to disseminate their messages [15,110]. Twitter “has become an important platform for eloquent and media-savvy people outside the traditional political, economic, or academic elites” [15]. Our study extends this line of research to intermedia agenda setting in vaccine-related conversations. The content of the URLs shared over Twitter represented, to a great degree, an alternative agenda. In this agenda, stories that advanced political motives that went beyond the issue of vaccination were featured prominently. They represented both opposition to Trump’s US presidential administration at the time of data collection and right-wing populist views, including vaccine-opposed content. Similarly, while some of the news coverage about vaccine development stood the test of time, a few stories that reported on vaccine advancement were inaccurate, such as the Israeli development of a vaccine. This type of coverage is likely to increase public doubt regarding news in general and scientific news in particular. Agenda setting by non-legacy media sources has both theoretical and practical implications for public health efforts. Unfortunately, in this context, these sources are also used by Twitter users associated with misinformation, conspiracy theories, and vaccine opposing messages.

In this new media environment, official health sources like the CDC and the NIH have had some success in disseminating their information, indicated by their inclusion in the lists of the most-tweeted domains (ie, CDC) and the most–consistently shared domains (ie, CDC and NIH). This is important, as governmental sources have been shown to provide credible, high-quality information compared to other sources, including the media [42]. However, producing the most-tweeted unique websites, in other words, “becoming viral,” proved more challenging, at least in the context of cross-platform sharing. The importance of trustworthy sources that provide scientifically sound information to the public is heightened at a time of a pandemic. Our findings revealed that the CDC had a salient role in the vaccine conversation on Twitter as the 20th most-tweeted website domain in our sample. The CDC was also second only to YouTube in providing a large number of different websites, which indicated that it provided diverse information that was deemed important by Twitter users who felt the need to share this information over Twitter. In comparison, no NIH-specific unique link to a website was included in the top 20 most-tweeted websites, but its domain was one of the 20 most–consistently tweeted domains. Although it is also possible that the CDC, NIH, and other public health sources exuded additional influence via links to traditional media shared over Twitter, such influence was not evident in the 20 most-tweeted websites.

Given the prominence of traditional media with their established gatekeeping, checks, and balances, it is not surprising that most stories shared in websites did not include obvious vaccine-opposing content. This finding is consistent with previous content analyses of tweets, reporting that vaccine-opposing content comprised a minority of the overall discussions on Twitter [16,111,112]. While this is encouraging from a public health perspective, it is important to remember that the impact of misinformation might still be significant, particularly in view of the social network nature of Twitter that often broadcasts to specific groups [36] and the need for herd immunity in maximizing the effects of vaccinations [112].

Our findings also point at the salience of international content. In addition to large, global media institutions like Reuters or CNN, and US-based newspapers and tabloids, some British newspapers, most notably The Guardian, were heavily tweeted in our data. Moreover, both the Israeli newspaper, The Jerusalem Post, and the Nigerian digital newspaper, The Cable, were included in list of the most-shared websites thanks to the viral story about an alleged Israeli COVID-19 vaccine. Similar to other smaller media organizations that were heavily tweeted, these two foreign, small newspapers demonstrated the opportunity of small players to advance their agenda in this new media environment by becoming “viral” through provision of sensational narratives. In the case of the Nigerian newspaper, inauthentic targeted attempts to spread this story contributed to its popularity, demonstrating the importance of deliberate manipulation in the new social media environment. It is likely that this rapidly changing environment, characterized by a “bursty” pattern of website sharing and the need to continuously provide new and sensational narratives as well as information and inauthentic spread strategies, poses unique challenges for governmental and official health sources. In addition, social media cross-platform sharing was evident in the prominence of YouTube as the third most-shared domain, as well as the most–consistently shared website over time. Future studies should further explore the content included in the different platforms, as well as users’ interpretation of this content and their motivation to engage in sharing it.

Finally, these findings are important in revealing patterns of propagation of this external content and these links. Despite the known presence of bots and other inauthentic propagation strategies of vaccination-related content on Twitter [8,37], and regarding COVID-19 [44], previous studies typically focused on analyzing the content of tweets in attempts to identify misinformation [45]. Such studies are important in advancing the knowledge and theories concerning the content to which users are exposed. However, the propagation strategies of this content should also be understood and considered. For instance, interventions to block such content should consider the propagation strategies. Given silos in current studies, social cybersecurity methods, to our knowledge, were not previously applied to vaccination-related discourse. Our study, therefore, is important in providing an opportunity to explore propagation of vaccine-related content over Twitter by spread of external content.

These results have important implications that can inform interventions, policies, and future research. At the most basic level, our findings indicate that sharing links to websites is a common strategy in Twitter conversations on the topic. In fact, shared websites were more common than hashtags, which have become synonymous with Twitter. Hashtags are frequently researched due to their use in creating discussion communities on social media [113,114]. It is, therefore, significant that in the context of the vaccine discussions we examined, external websites were featured as frequently as hashtags.

Our analysis also revealed that websites were tweeted in a “bursty” pattern, indicating heterogeneity of a large number of sources, stories, and topics shared. The results regarding the increased number and diversity of external links shared at the time of data collection are consistent with other studies that documented the ebbs and flows of the “infodemic.” A recent study suggested that this increase was motivated by both uncertainty and state-sponsored propaganda [115]. COVID-19 is used as vector to propagate misinformation and disinformation by foreign governments. In addition, it provides a highly uncertain information environment in which fact-checking is difficult. The authors emphasized the importance of constant, reliable medical information provided by governmental sources. While we concur with this suggestion, it is important to note that our findings also point at the challenges of such public health response. Given the numbers, diversity of sources, and dynamics of the topic that are constantly evolving, such response would require significant efforts and resources [115].

### Strengths and Limitations

The strengths of this study stem from its analysis of a large data set that was collected at a historically important period. Moreover, we employed a triangulation of computational methods and human coding to study a previously unexplored communication strategy within vaccine discourse on Twitter. However, this study is not without limitations. First, our tweets were collected by searching common vaccine-related keywords and hashtags. While these keywords and hashtags were identified following an extensive literature review and analysis of tweets by multiple research teams, it is possible that some emerging keywords and hashtags were not included. Future studies could apply additional computational methods, such as the Analysis of Topic Model Networks [116].

Additional limitations are grounded in our focus on tweets in English and on a specific time frame. Future studies should expand research to include additional languages and time frames, particularly during and following the rollout of the COVID-19 vaccine. In addition, some vaccine-related tweets, particularly those advancing vaccine-opposing messages, were deleted by Twitter by the time of analysis. Hence, the actual number of antivaccination tweets shared might be higher than what we were able to report, and their content might be somewhat different from what was collected. Moreover, we have focused on vaccine-related tweets that were part of the COVID-19 Twitter conversations. Although our data set is unique in including all related tweets rather than a sample, our findings do not apply to vaccine-related discourse on Twitter that was not part of the pandemic discourse. Moreover, our study focused on the first 20 weeks of the pandemic. Future studies should compare our findings regarding website sharing with similar content following the implementation of the COVID-19 vaccination campaigns globally. In addition, we focused on the content and propagation of the 20 most-tweeted websites and domains, and these findings might not apply to other links shared in this data set.

### Conclusions

These findings are important in advancing understanding of website sharing in vaccine-related tweets, its use, and its dynamics. The analysis revealed that Twitter users share websites as part of their vaccine messages in COVID-19 conversations and that some of this sharing revealed inauthentic, deliberate attempts to spread this content. Our data included tweets that were posted in the first 5 months of the pandemic and showed that vaccine-related tweets were prominent in the pandemic-related Twitter discourse from its inception. Future research should examine the following months, as it is likely that with the advances in vaccine development, these conversations have increased in frequency and perhaps included different information sources.

The findings of this study pave the way for future studies that would answer additional questions. First and foremost, future research should expand the scope of this study by examining websites shared after June 2020, especially as new COVID-19 vaccines were approved and disseminated, and as information became available about their safety and efficacy. Given that our findings encompass the period prior to the approval and dissemination of the specific COVID-19 vaccines, they shed light on early communication on the topic rather than the specific risks and benefits of these vaccines.

In view of the global importance of the pandemic and vaccinations, future studies should also expand the scope of analysis to include additional languages other than English. In addition, it is important to consider, measure, and analyze additional aspects and implications of our work. For instance, to date, studies did not explore the impact of visual content on vaccine-related messages over social media. Future studies should expand the scope of this study’s analysis by exploring the visual content of vaccine-related tweets, websites, and of YouTube videos on the topic and the visual impact on propagation of the content over social networks. Similarly, future studies should explore the content and propagation of additional URLs in addition to the 20 most-tweeted websites explored in this study.

Finally, we call for hypothesis-driven communication interventions that would not only measure how and why antivaccination messages propagate over social media [117] and attempt to correct misinformation [118], but would also attempt to prevent this propagation and advance scientifically accurate content instead. Such future interventions should not focus on one social media platform, but should instead consider and integrate cross-platform use for message sharing.

## Abbreviations

API: application programming interface
CDC: Centers for Disease Control and Prevention
mRNA: messenger RNA
NIH: National Institutes of Health
WHO: World Health Organization
