# Correction: Content and Dynamics of Websites Shared Over Vaccine-Related Tweets in COVID-19 Conversations: Computational Analysis

**DOI:** 10.2196/43279

**Published:** 2023-01-09

**Authors:** Iain Cruickshank, Tamar Ginossar, Jason Sulskis, Elena Zheleva, Tanya Berger-Wolf

**Affiliations:** 1 Center for Computational Analysis of Social and Organizational Systems Carnegie Mellon University Pittsburgh, PA United States; 2 Department of Communication and Journalism University of New Mexico Albuquerque, NM United States; 3 Department of Computer Science The University of Illinois at Chicago Chicago, IL United States; 4 Translational Data Analytics Institute The Ohio State University Colombus, OH United States

In “Content and Dynamics of Websites Shared Over Vaccine-Related Tweets in COVID-19 Conversations: Computational Analysis” ([JMIR Res 2021;23(12):e29127) the authors made one clarification.

The sentences:

Whereas the exact identity of the alternative media that was documented in this study (e.g., Raw Story) might not necessarily be important in a historical perspective, these processes of agenda setting have both theoretical and practical implications for public health efforts. Unfortunately, in this context, prominence of these fringe sources is also associated with misinformation, conspiracy theories, and vaccine-opposing messages.

Have been clarified to read:

Agenda setting by non-legacy media sources has both theoretical and practical implications for public health efforts. Unfortunately, in this context, these sources are also used by Twitter users associated with misinformation, conspiracy theories, and vaccine opposing messages.

The correction will appear in the online version of the paper on the JMIR Publications website on January 9, 2023, together with the publication of this correction notice. Because this was made after submission to PubMed, PubMed Central, and other full-text repositories, the corrected article has also been resubmitted to those repositories.

